# Knocking down *Pseudomonas* *aeruginosa* virulence by oral hypoglycemic metformin nano emulsion

**DOI:** 10.1007/s11274-022-03302-8

**Published:** 2022-05-30

**Authors:** Salwa E. Gomaa, Ghada H. Shaker, Farag M. Mosallam, Hisham A. Abbas

**Affiliations:** 1 Department of Microbiology and Immunology, Faculty of Pharmacy, Zagazig University, Zagazig, Egypt; 2 Drug Microbiology Lab., Biotechnology Division, Drug Radiation Research Department, National Center for Radiation Research and Technology (NCRRT), Egyptian Atomic Energy Authority, Cairo, Egypt

**Keywords:** *Pseudomonas aeruginosa*, Metformin, Silver, Nanoformulations, Quorum sensing, Virulence inhibition

## Abstract

Long-term antibiotic treatment results in the spread of multi-drug resistance in *Pseudomonas aeruginosa* that complicates treatment. Anti-virulence agents can be viewed as alternative options that cripple virulence factors of the bacteria to facilitate their elimination by the host immunity. The use of nanoparticles in the inhibition of *P. aeruginosa* virulence factors is a promising strategy. This study aims to study the effect of metformin (MET), metformin nano emulsions (MET-NEs), silver metformin nano emulsions (Ag-MET-NEs) and silver nanoparticles (AgNPs) on *P. aeruginosa* virulence factors’ expression. The phenotypic results showed that MET-NEs had the highest virulence inhibitory activity. However, concerning RT-PCR results, all tested agents significantly decreased the expression of quorum sensing regulatory genes of *P. aeruginosa*; *lasR, lasI, pqsA, fliC, exoS* and *pslA*, with Ag-MET-NEs being the most potent one, however, it failed to protect mice from *P. aeruginosa* pathogenesis. MET-NEs showed the highest protective activity against pseudomonal infection in vivo. Our findings support the promising use of nano formulations particularly Ag-MET-NEs as an alternative against multidrug resistant pseudomonal infections via inhibition of virulence factors and quorum sensing gene expression.

## Introduction

*Pseudomonas aeruginosa* is a frequent etiological agent of opportunistic and hospital acquired infections (Pang et al. 2019). *P. aeruginosa* infection may trigger severe complications in immunocompromised patients and those suffering from respiratory, urinary tract and burn infections, sepsis, cystic fibrosis, osteomyelitis and endocarditis (Moradali et al. 2017). Several virulence factors mediate the pathogenicity of *P. aeruginosa* such as pili, flagella, pyocyanin, pyoverdin, elastase, hemolysins, proteases, rhamnolipids, exotoxin A and biofilm formation (Lee and Zhang 2015; Veesenmeyer et al. 2009). The overuse of antibiotics has contributed to the spread of multidrug resistant *P. aeruginosa* (MDR) infections (Aloush et al. 2006). Synthesis of new antibiotics is time-consuming and requires high cost-effectiveness. Moreover, rapid resistance advances shorten their lifetime (Boucher et al. 2013; Fernandes and Martens 2017). Therefore, new therapeutic approaches are required to tackle the problem of MDR organisms. One useful approach is anti-virulence therapy using FDA approved drugs (drug repurposing). This approach has the advantages of disarming of pathogens without killing them, and the availability of data on their safety and pharmacokinetics. This decreases the economic costs as well as the time needed for the process of drug development (Finlay and Falkow 1997; Miró-Canturri et al. 2019; Mullard 2012; Rasko and Sperandio 2010).

The capacity of *P. aeruginosa* to form biofilm is a major virulence factor. The biofilm forming cells are more tolerant to the antibiotics and the host immune system (Whiteley et al. 2001). In addition, *P. aeruginosa* produces pyocyanin pigment that interacts with molecular oxygen forming hydrogen peroxide (H_2_O_2_) and other reactive oxygen species (ROS). This leads to altered redox balance of the host tissues leading to the injury of cells and may lead to death (Price-Whelan et al. 2006). *P. aeruginosa* also produces proteases that degrade the host lung elastin resulting in lung damage which occurs during respiratory tract infections, particularly in patients with chronic cystic fibrosis (Kipnis et al. 2006). *P. aeruginosa* shows motility in one of three forms namely; swimming, swarming in addition to twitching motility (Floyd et al. 2016). Swarming cells in *P. aeruginosa* contribute to biofilm formation, antibiotic resistance and overexpression of numerous virulence factors (Coleman et al. 2021).

Quorum sensing ‘intercellular signaling network’ is the main regulator of bacterial virulence. This can occur through bacterial secretion of auto-inducers or signaling molecules, their concentration is directly proportional to the bacterial cell density. After reaching a maximal concentration, genes encoding virulence factors are activated (Davies et al. 1998). *P. aeruginosa* has four interacting QS signaling systems; *las* system and *rhl* system which depend on the secretion and recognition of N-acyl-homoserine lactone autoinducers (AHL), in addition to the *Pseudomonas* quinolone signal (*PQS*) system, and the integrated QS (*IQS*) system (Lee and Zhang 2015). This network of cell to cell communication gives *P. aeruginosa* the capacity to produce extracellular virulence factors merely at threshold concentration (Van Delden and Iglewski 1998).

The use of nanotechnology is required to overcome the global dilemma of bacterial resistance to antimicrobials (Wang et al. 2017). Nanomaterials vary between 1 and 100 nm in size and display distinct physical and chemical properties compared to their bulk matter (Wang et al. 2017). The use of materials in nanomeric size leads to greater interaction between bacteria and compound, eases their penetration into the cell, increases absorption and improves bioavailability (Jamil and Imran 2018; Zaidi et al. 2017). Nanomaterials can be synthesized by many methods such as chemical, physical in addition to biological methods (Kaur 2018). The radiation induced synthesis of nanomaterials has several advantages over the traditional methods, because of its simplicity, no need for excess reducing agents and no excessive oxidation products. In addition, products are completely reduced and present in a highly stable pure state (Remita et al. 1996).

Metformin is regarded as one of the most common oral hypoglycemics used for treatment of patients with type 2 diabetes mellitus. Chemically, metformin belongs to the biguanide moiety of drugs that is especially useful for obese patients (Essmat et al. 2020). Metformin disrupts the membrane permeability of bacteria. In addition, it can compromise bacterial cell walls enhancing the antibacterial activity of antibiotics by increasing their intracellular accumulation. Moreover, they modify the immune response leading to increasing resistance to infection (Coates et al. 2020; Liu et al. 2020; Xiao et al. 2020). In addition, it was reported to exhibit anti-virulence activity by interfering with quorum sensing that regulates the production of virulence factors such as biofilm, proteases, pyocyanin, elastase and hemolysin production of *P. aeruginosa* PAO1 in a study done by Abbas et al. (2017).

Since ancient times, metals have been used as antibacterial agents. Silver is one of the most widely used. This is attributed to its powerful antimicrobial activity as well as its low toxicity (Chen and Schluesener 2008). Silver nanoparticles (AgNPs) exhibit activity against various type of microorganisms such as viruses, bacteria and fungi (Murphy et al. 2015). Silver nanoparticles can be seen as the most effective nanomaterial that can be used against MDR bacteria, however, other metallic NPs like AuNPs, CuONPs, Fe^3^O^2^NPs and TiONPs show good activity (Dakal et al. 2016; Hemeg 2017; Slavin et al. 2017). In addition, several studies reported the anti-virulence activity of nanomaterials (Li et al. 2020; Loo et al. 2016; Qais et al. 2021).

The cytotoxic effects of AgNPs, documented in vitro studies in various cell lines, are governed by factors such as size, shape, coating, dose and cell type. In addition, toxicity and biodistribution studies, in vivo, following various routes of exposure, like inhalation, instillation, oral, dermal and intravenous, have established Ag translocation, accumulation, and toxicity to various organs (Ferdous and Nemmar 2020).

The current study aimed to evaluate the possible quorum sensing inhibitory activity of metformin and to determine for the first time whether it can be more effective in nanoform than in bulk. In addition, investigate if a combination of metformin with AgNPs may help in attenuation of *P. aeruginosa* virulence and pathogenicity.

## Materials and methods

### Media and chemicals

Mueller hinton agar (MHA) and mueller hinton broth (MHB), tryptone soya agar (TSA), tryptone soya broth (TSB), and MacConkey agar were obtained from Oxoid (St. Louis, USA). Other chemicals were of pharmaceutical grade. Metformin (MET) and silver nitrate were purchased commercially from Sigma Chemical Company, St. Louis, Mo, USA.

### Bacterial isolates

The standard strain *P. aeruginosa* ATCC 27853 was used in this study. It was provided from the stock culture collection of Microbiology and Immunology Department, Faculty of medicine, Zagazig University. Six clinical MDR *P. aeruginosa* isolates (PA1, PA2, PA3, PA4, PA5 and PA10) were obtained from the stock culture collection of Microbiology and Immunology Department, Faculty of pharmacy, Zagazig University. They were obtained from patients with burn, surgical wound, respiratory tract and urinary tract infections. All isolates were maintained in MHB with 10–15% glycerol and kept at − 80 °C.

### Metformin nano emulsionss and silver metformin nano emulsions preparation

In order to prepare both metformin nano emulsion (MET-NEs) and silver metformin nano emulsions (Ag-MET-NEs), the modified ultra-sonication method was used as referenced (Laxmi et al. 2015; Mosallam et al. 2021a).

In synthesis of MET-NEs and Ag-MET-NEs (O/W 30/70), both coconut oil oily phase and tween 80 emulsifier were added drop wise to aqueous phase of either MET in a concentration of 100 mg/mL or MET and already prepared AgNPs (100–0.05 mg/mL) using homogenizer at 10,000 rpm for 30 min for continuous stirring. Then, the ultrasonic sonicator was used to sonicate the emulsion for 1 h. For characterization of the prepared nano emulsions, different physicochemical parameters such as particle size and distribution in addition to zeta potential were measured at the National Center for Radiation Research and Technology (NCRRT), Cairo, Egypt. The charge of the particles determines the stability of the nano emulsions. Zeta potential was used to quantify the particle charge and it is detected by using the electrophoretic motion of the particles in an electrical field. DLS Zeta Sizer Technique (PSS-NICOMP 380-ZLS, USA) was used to measure zeta potential of the optimized formulation. The particle sizes of the prepared nano emulsions were performed by Transmission Electron Microscopy (TEM) using (JEOL electron microscope JEM-100 CX) at an accelerating speed of 80 kV.

### Determination of minimum inhibitory concentrations (MICs) of the tested agents

The minimum inhibitory concentrations (MICs) of MET, MET-NEs (stock solution of 100 mg/mL, each), Ag-MET-NEs (100–0.05 mg/mL) and AgNPs (0.05 mg/mL) against *P. aeruginosa* were assessed using the broth micro-dilution method using 96-well microtiter plate according to the clinical and laboratory standards institute (CLSI) guidelines (Wikler 2006).

### Phenotypic assay of *P*. *aeruginosa* virulence factors

#### Bioflm inhibition assay

The capacity of the tested clinical strains to produce biofilm was quantitatively assayed according to the method previously described by Abbas et al*.* (2017). The standard strain *P. aeruginosa* ATCC 27853 was previously reported to have strong biofilm forming capacity (Casciaro et al. 2019).

To test the inhibitory activity of tested agents against biofilms, the same procdure was repeated in the presence of 1/10 MIC of them. The following formula was used in order to calculate the biofilm inhibitory percentage (%);

$$\% \,{\text{of}}\,{\text{biofilm}}\,{\text{inhibition}}\, = \,{\text{Abs}}\,{\text{of}}\,{\text{control}}\,{\text{at}}\,{\text{OD}}\,{\text{59}}0\,{\text{nm}} - \,{\text{Abs}}\,{\text{of}}\,{\text{test}}\,{\text{at OD 59}}0{\text{ nm}}/{\text{Abs of control at OD 59}}0{\text{ nm }} \times {\text{ 1}}00$$

#### Pyocyanin inhbition assay

The inhibitory activities of the tested agents against pyocyanin was assessed according to the method described by Das and Manefield (2012).

#### Swarming motility inhbition assay

In order to test the capacity of the tested agents to block the swarming motility of *P. aeruginosa* isolates, Krishnan et al*.* method was performed (Krishnan et al. 2012).

#### Total proteases inhbition assay

The effect of the tested agents on inhibition of total proteases by *P. aeruginosa* isolates was carried out using the modified skimmed milk broth method. *P. aeruginosa* overnight cultures in MHB with and without 1/10 MIC of the tested agents were centrifuged to obtain the supernatants. Aliquots of 500 μL of bacterial supernatants were incubated with 1 mL skimmed milk (1.25%) for 1 h at 37 °C. The decrease in optical density of skimmed milk was estimated at 600 nm using Biotek spectrofluorometer (USA) and considered as measure of proteolytic activity. The test was performed twice (El-Mowafy et al. 2014).

### Assessment of the effect of the tested agents on the expression of some virulence genes in *P*. *aeruginosa* using qRT‑PCR

The ability of the tested drugs to downregulate the expression of QS controlled genes; namely *lasR, lasI, pqsA, fliC, pslA* and *exoS* in the standard strain *P. aeruginosa* ATCC 27853 was assessed by qRT-PCR. The total bacterial RNA extract was purified using TRIzol Reagent (15596026, Life Technologies, USA) according to the manufacturer instructions. In order to synthesize cDNA, QuantiTect Reverse Transcription Kit was used and it was amplified by Thermo Scientific Maximas SYBR Green/Fluorescein qPCR Master Mix. The primers used are presented (Table 1). The relative expression level of the tested genes was normalized to the housekeeping gene (*rpoD*) using the 2^−ΔΔCt^ method (Livak and Schmittgen 2001). The experiment was performed in triplicate.

**Table 1:** List of *P*. *aeruginosa* primers used in qRT-PCR

Gene	Sequence of primers	References
*lasI*	F/5′-CGCACATCTGGGAACTCA-3′ R/5′-CGGCACGGATCATCATCT-3′	El-Mowafy et al. (2014)
*lasR*	F/5′-CTGTGGATGCTCAAGGACTAC-3′ R/5′-AACTGGTCTTGCCGATGG-3′	El-Mowafy et al. (2014)
*pqsA*	F/5′-GACCGGCTGTATTCGATTC-3′ R/5′-GCTGAACCAGGGAAAGAAC-3′	El-Mowafy et al. (2014)
*fliC*	F/5′-GCTTCGACAACACCATCAAC-3′ R/5′-AGCACCTGGTTCTTGGTCAG-3′	Roberts et al. (2015)
*exoS*	F/5′-CCATCACTTCGGCGTCACT-3′ R/5′-GAGAGCGAGGTCAGCAGAG-3′	El-Mowafy et al. (2014)
*pslA*	F/5′-TCCCTACCTCAGCAGCAAGC-3′ R/5′-TGTTGTAGCCGTAGCGTTTCTG-3′	El-Demerdash and Bakry (2020)
*ropD*	F/5′-CGAACTGCTTGCCGACTT-3′ R/5′-GCGAGAGCCTCAAGGATAC-3′	El-Mowafy et al. (2014)

*F* forward, *R* reverse

### Evaluation of the efficacy of the tested agents on pathogenicity in mice model

The effect of the tested agents on the pathogenicity of *P. aeruginosa* ATCC 27853 was assessed by using mice as an infection animal model. The experment was performed in compliance to the local guidelines for animal welfare approved by the committee of The Institutional Animal Care and Use, Zagazig University (ZU-IACUC), Egypt (Approval number: ZU-IACUC/3/F/114/2020). The bacterial burden in mice was detected as previously reported by Deshmukh et al*.* with some modifications (Deshmukh et al. 2009). Overnight cultures of *P. aeruginosa* in MHB with and without 1/10 MIC of the tested agents were prepared. The cultures were centrifuged and the pellets were resuspended in buffered saline (PBS) to reach cell density equal to 2.5 × 10^7^ CFU/mL. Seven random groups of 5–6 weeks old healthy albino mice (*Mus musculus*) with equal weights were included in the experiment. Each group consists of five mice was used for *P. aeruginosa*. In group 1, untreated bacteria in sterile PBS (100 µL) were used for intraperitoneal injection of the mice. In group 2, 100 µL of bacteria treated with MET were injected in mice, group 3 was injected with 100 µL of MET-NEs-treated bacteria, while group 4, was injected with 100 µL of Ag-MET-NEs-treated bacteria and group 5 was injected with 100 µL of AgNPs-treated bacteria. Two additional groups were used as negative controls; group 6, mice were intraperitoneally injected with sterile PBS (100 µL), while in group 7, mice were uninoculated. Normal feeding and aeration were given to all groups at room temperature. After 24 h postinfection, mice were anaesthetized, sacrificed, livers and kidneys were harvested, weighed and homogenized for enumeration of live bacterial cells as colony forming unit per gram (CFU/g).

### Statistical analysis

The inhibitory activity of the tested agents against virulence factors was analyzed using GraphPad Prism 8 software (One Way ANOVA followed by Dunnett’s multiple comparison tests or Bonferroni’s multiple comparison test) at *P* < 0.05 for signifcance.

## Results

### Synthesis and cheracterization of metformin nano emulsions and silver metformin nano emulsions

**Fig. 1:**
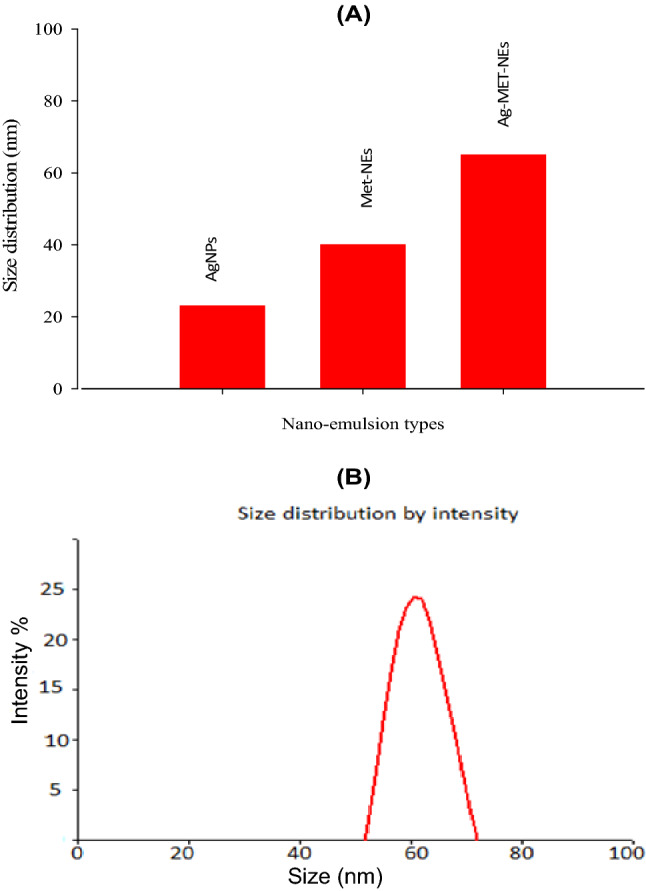
**A)** Size distribution diagram of AgNPs, MET-NEs, Ag-MET-NEs and **B)** DLS image of Ag-MET-NEs

**Fig. 2:**
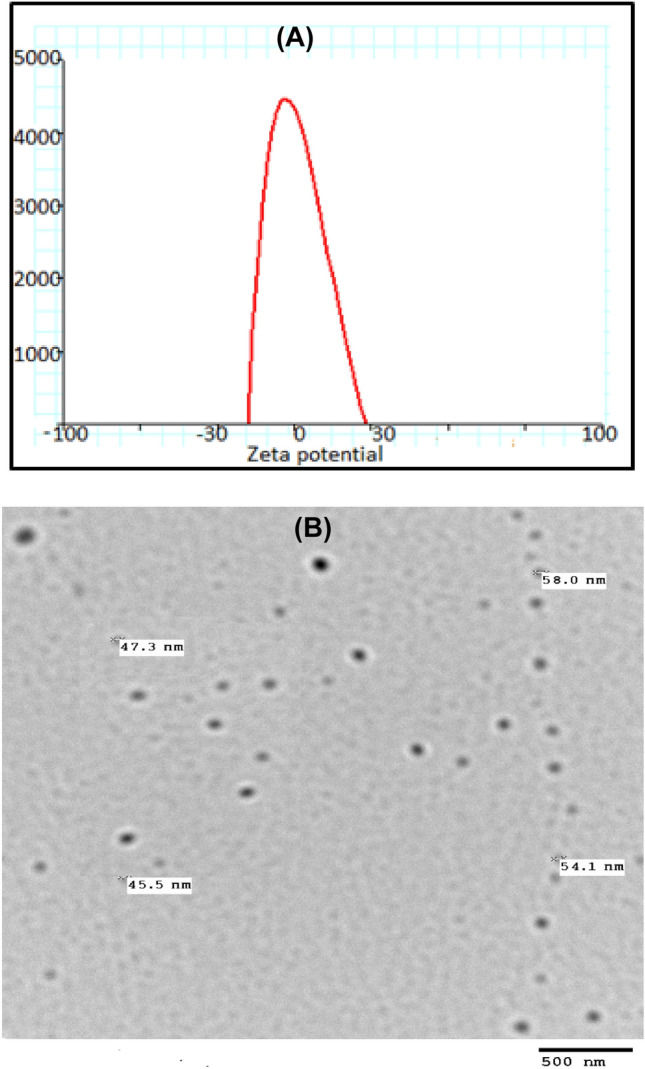
**A)** Zeta potential of Ag-MET-NEs and **B)** TEM image of Ag-MET-NEs

An important idiosyncratic property of nanoemulsion is its nanoscale particle size. The size distribution analysis of MET-NEs and Ag-MET-NEs was performed using DLS Zeta Sizer Technique. The illustration of the comparative particle size distribution of initially prepared AgNPs, MET-NEs and Ag-MET-NEs is shown in Fig. 1a. Figure shows size distribution with 23, 40 and 65 nm, respectively. Moreover, Fig. 1b shows the particle size distribution (DLS) of Ag-MET-NEs with 65 nm. Figure 2a shows the zeta potential at range from − 30 to 30 mV.

Figure 2b shows the TEM image of Ag-MET-NEs that confirms the circle shape of particles with average size of about 52 nm. The presence of metformin serving as capping and tween as stabilizing agents controls and prevents the aggregation and agglomeration of generated NPs.

### Minimum inhibitory concentrations (MICs) of the tested agents against *P*. *aeruginosa*

Minimum inhibitory concentrations (MICs) were determined using the broth microdilution method. There was no difference between the MICs of MET and MET-NEs against the tested bacteria. However, the MICs were markedly lowered upon using the combination of MET and AgNPs (Ag-MET-NEs) compared with either MET, MET-NEs or AgNPs alone. Considering the increase in sensitivity to either MET or MET-NEs, the MICs were decreased by 16 to 128 folds, while for AgNPs (16–64) folds among the tested isolates (Table 2). The activity of the tested agents against quorum sensing and virulence of the tested isolates was evaluated at 1/10 MIC.

**Table 2:** MIC values of the tested agents against *P*. *aeruginosa*

Tested isolates	MET (100 mg/mL)	MET-NEs (100 mg/mL)	Ag-MET-NEs (100–0.05 mg/mL)	AgNPs (0.05 mg/mL)
PA1	50	50	1.56	0.025
PA2	50	50	1.56	0.0125
PA3	50	50	1.56	0.025
PA4	50	25	1.56	0.025
PA5	50	50	1.56	0.025
PA10	50	50	1.56	0.025
PA ATCC 27,853	50	50	0.39	0.0125

*MET* metformin, *MET-NEs* metformin nano emulsion, *Ag-MET-NEs* silver metformin nano emulsion, *AgNPs* silver nanoparticles

### Phenotypic inhibition of virulence factors of *P*. *aeruginosa* by the tested agents

#### The tested agents inhibited biofilm formation

**Fig. 3:**
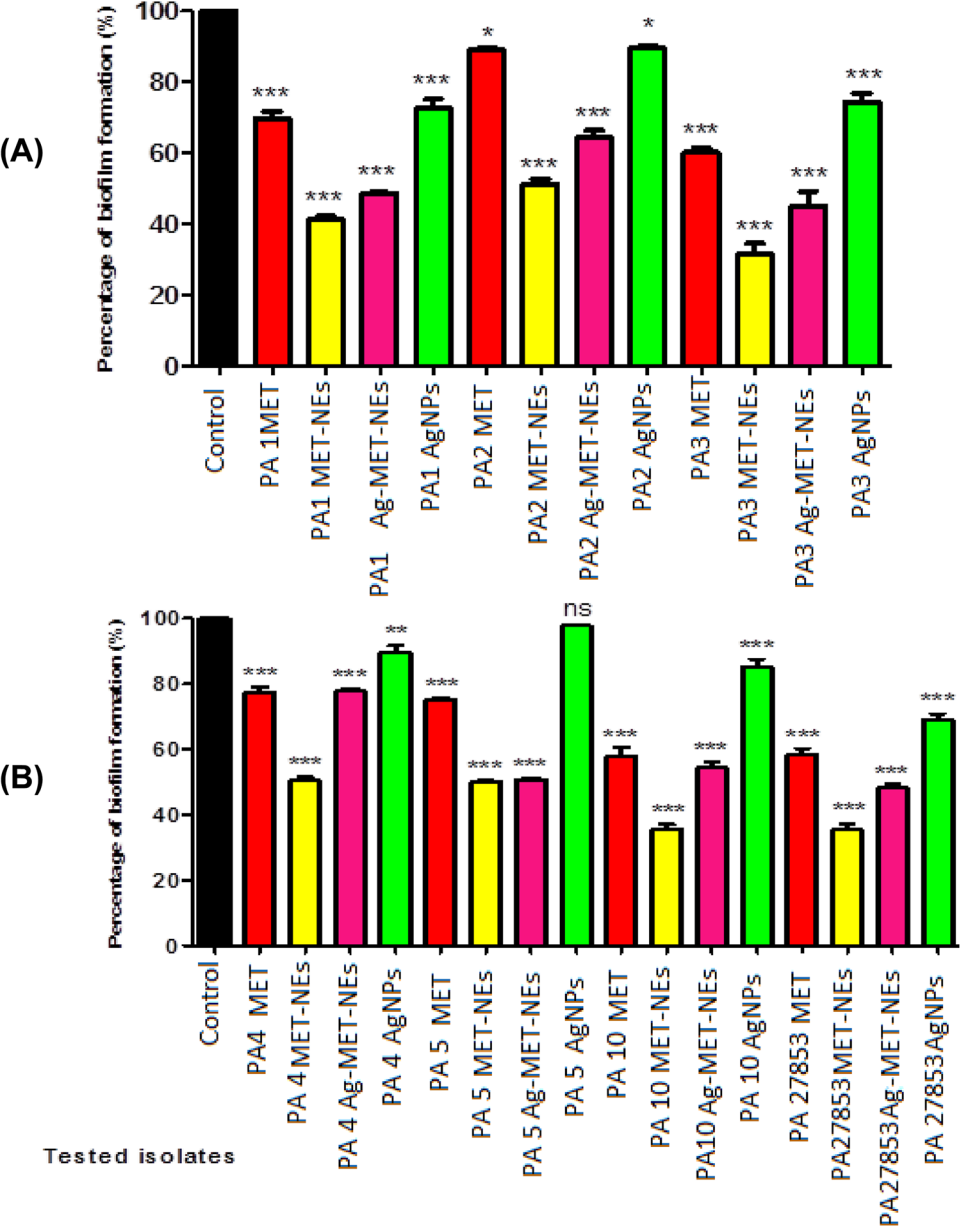
Inhibition of biofilm formation in *P*. *aeruginosa* by sub-MICs of the tested agents. **A**) Isolates PA1, PA2, and PA3, **B**) Isolates PA4, PA5, and PA27853 standard strain. Significant reduction in the biofilm formation was detected with 1/10 MIC of the tested agents against the tested isolates as compared to controls. Optical density was measured at 590 nm. The data shown represent the means ± standard errors. *Significant *P* < 0.05, ns non-significant

The biofilm inhibitory activities of the tested agents against *P. aeruginosa* were performed using crystal violet assay. The tested agents showed significant reduction in the biofilm formation compared to the control untreated isolates at (*P* < 0.05) as shown in (Fig. 3). A higher biofilm removal efficiency was found with MET-NEs (48.75–68.60%) and Ag-MET-NEs (22.09–54.84%) than MET (11.04–41.93%) and AgNPs (10.51–30.89%). The biofilm formation capacity of AgNPs was not significantly reduced in one isolate (PA5).

#### The tested agents decreased pyocyanin production

**Fig. 4:**
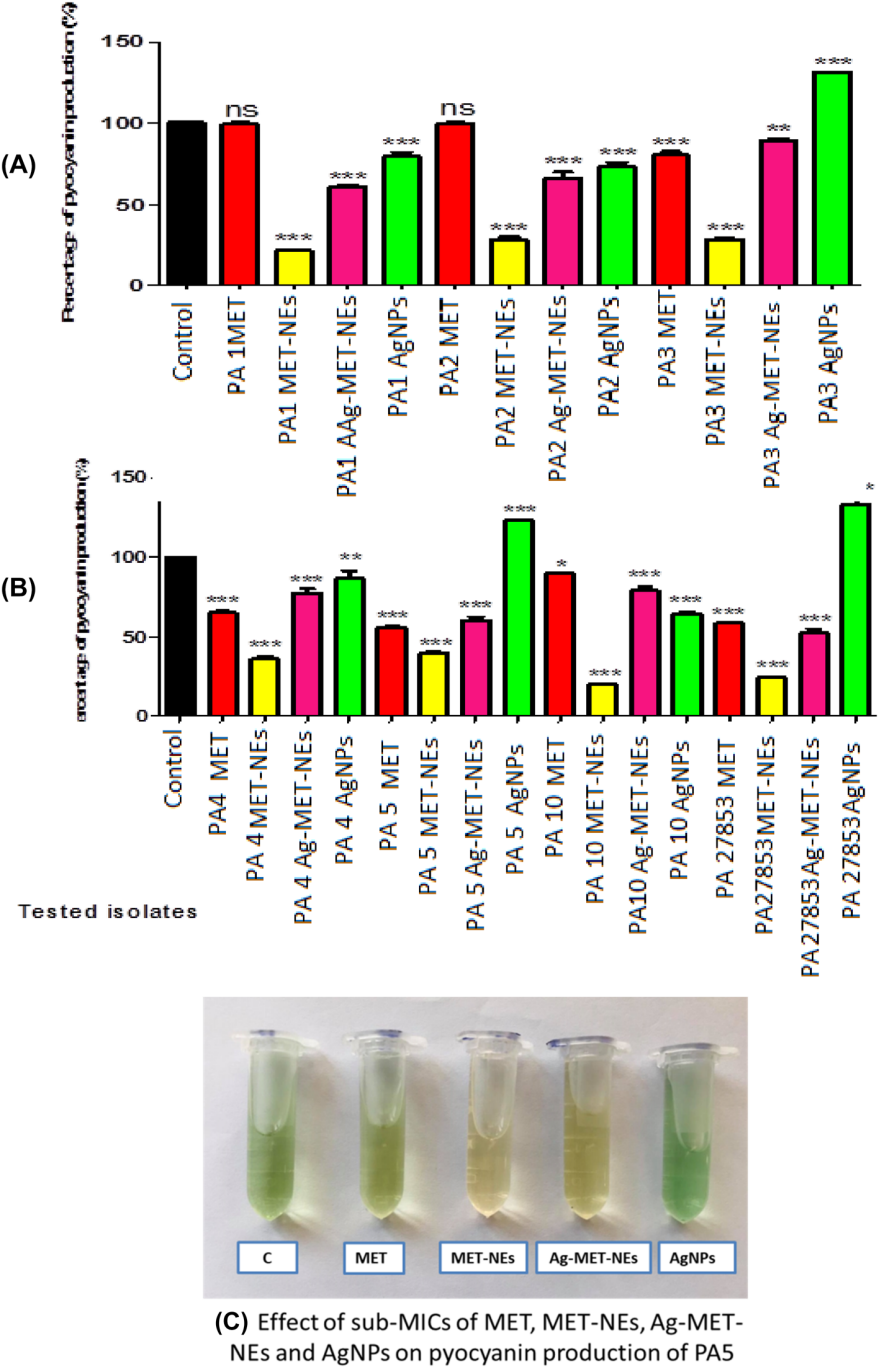
Effect of sub-MICs of the tested agents on pyocyanin production in *P*. *aerugino*sa. **A**) Isolates PA1, PA2, and PA3, **B**) Isolates PA4, PA5, and PA27853 standard strain, **C**) A representative image showing the effect of tested agents on pyocyanin. Pyocyanin pigment was measured at 691 nm, significant decline in production of pyocyanin pigment was observed in treated and untreated cultures. The data shown represent the means ± standard errors. *Significant *P* < 0.05, ns non-significant

The effect of the tested agents on pyocyanin production of *P. aeruginosa* was estimated spectrophotometrically. The tested agents showed significant reduction in pyocyanin production compared to the untreated controls at (*P* < 0.05) as shown in (Fig. 4). MET-NEs showed the highest inhibitory activity against pyocyanin production (60.01–79.99%). However, the inhibitory activities of Ag-MET-NEs, MET and AgNPs were lower; 10.59–47.78%, 0.24–44.10% and 13.39–35.28%, respectively. No significant reduction in pyocyanin pigment by MET was observed in two isolates (PA1 and PA2). AgNPs showed significant increase in pyocyanin production in PA3, PA5 as well as the standard strain.

#### The tested agents reduced swarming motility

**Fig. 5:**
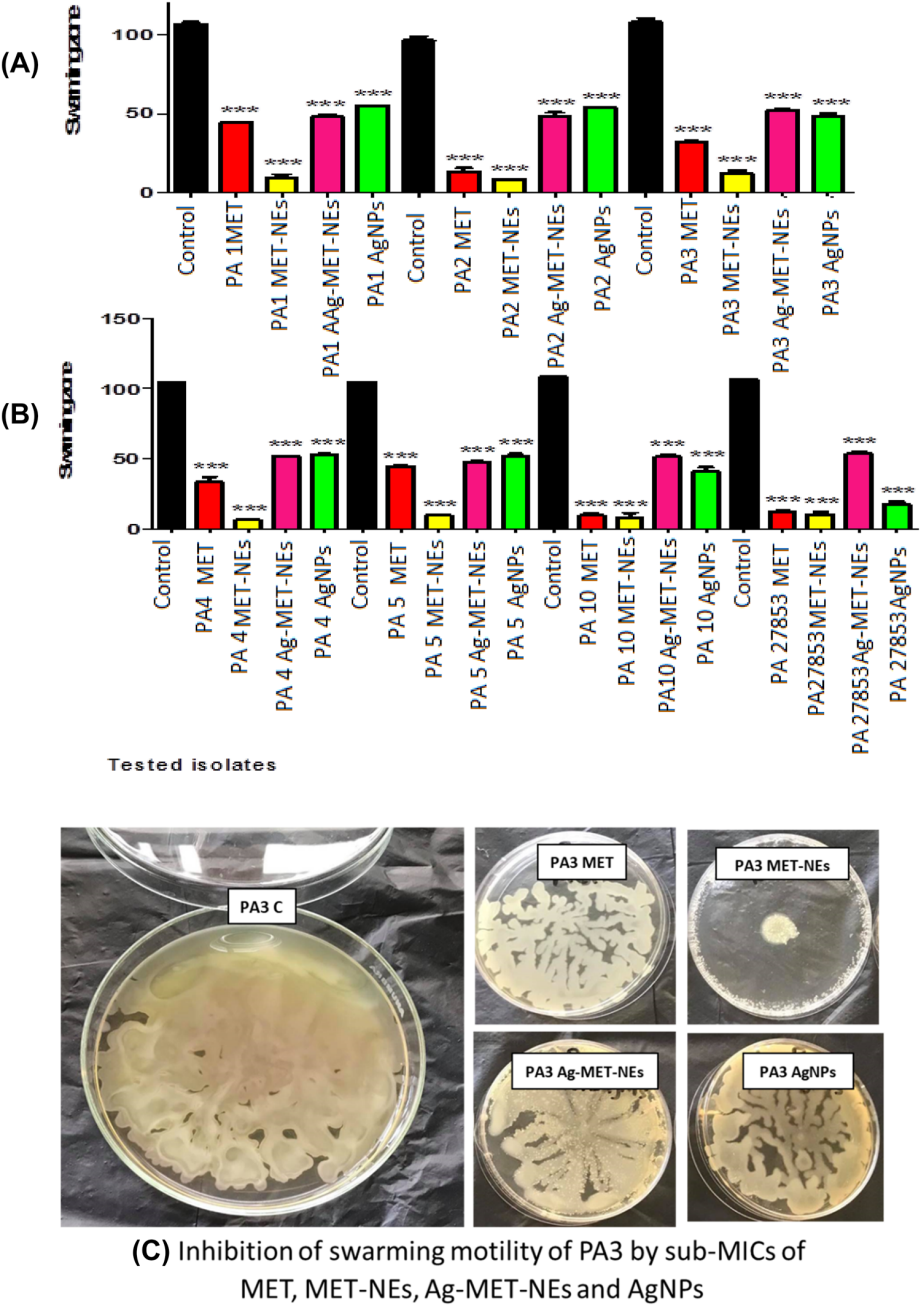
Inhibition of swarming motility in *P*. *aeruginosa* by sub-MICs of the tested agents. **A**) Isolates PA1, PA2, and PA3, **B**) Isolates PA4, PA5, and PA27853 standard strain, **C**) A representative image showing the effect of tested agents on swarming motility. Significant reduction in swarming motility of all tested bacteria with 1/10 MIC as compared to controls. The data shown represent the means ± standard errors. *Significant *P* < 0.05

The presence of sub-MICs concentration (1/10 MIC) of the tested agents significantly affected the swarming motility of all treated bacteria as compared to the untreated controls at (*P* < 0.05) as shown in (Fig. 5). MET-NEs showed maximum inhibition of swarming motility (88.87–94.16%) followed by MET (58.59–92.62%). Whereas, the inhibitory activities of Ag-MET-NEs (49.77–56.12%) and AgNPs (43.98–83.82%) were more or less similar.

#### The tested agents decreased total proteases

**Fig. 6:**
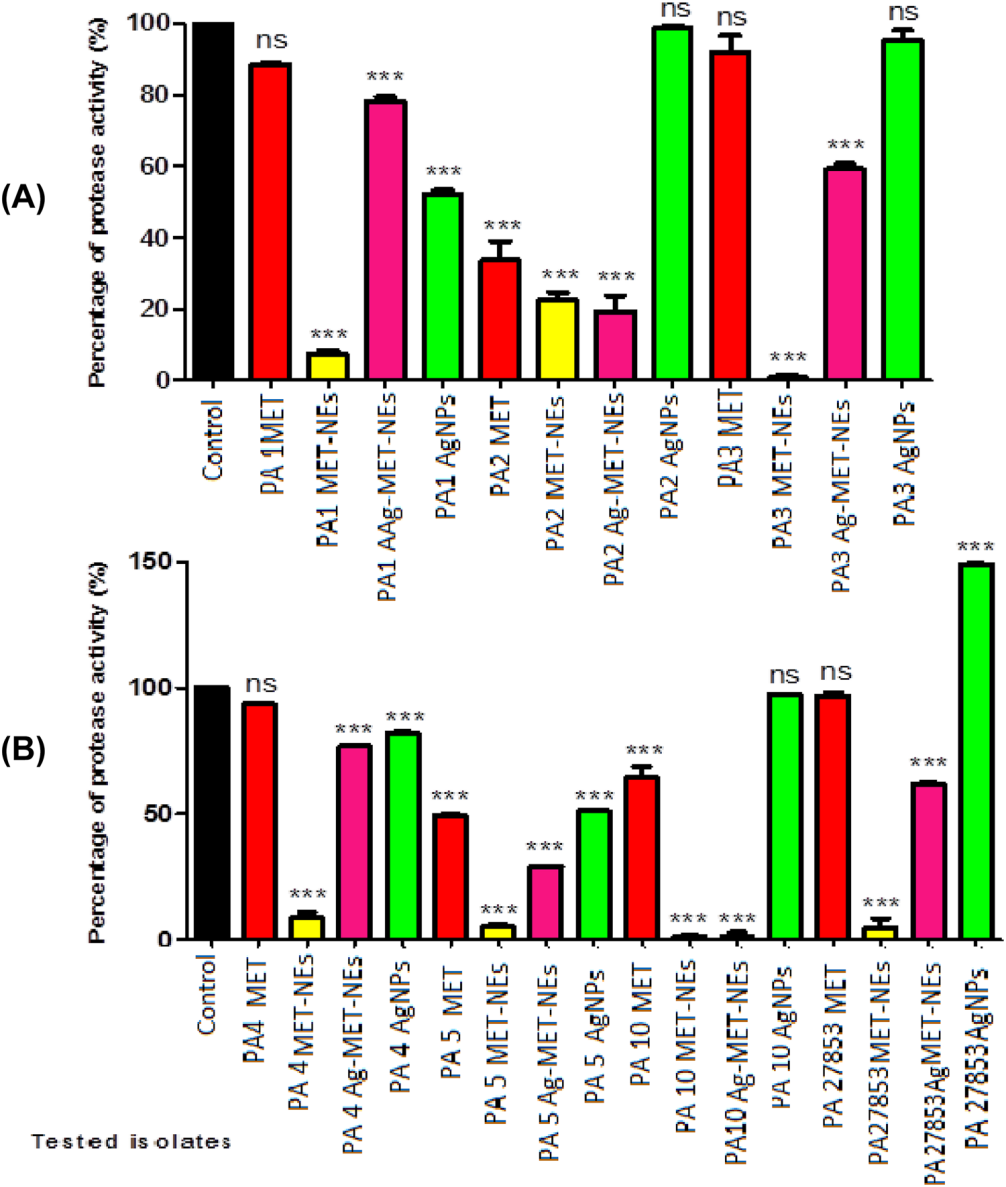
Effect of 1/10 MICs of the tested agents on levels of protease. **A**) Isolates PA1, PA2, and PA3, **B**) Isolates PA4, PA5, and PA27853 standard strain. OD_600_ was measured after overnight culturing of bacteria in MHB with and without 1/10 MICs of the tested agents followed by incubation of supernatants with skim milk for 1 h at 37 °C. The data shown represent the means ± standard errors. *Significant *P* < 0.05, ns non-significant

The ability of the tested agents to inhibit proteolytic activity was measured using the modified skimmed milk broth method. It was found that the inhibitory activity of MET-NEs (77.48–99.15%) was higher than MET (35.48–66.32%). Also, Ag-MET-NEs (21.76–98.52%) exhibited higher proteolytic activity than AgNPs (17.90–48.88%). No significant inhibition of protease production was observed with either MET or AgNPs in some tested bacteria. However, AgNPs increased protease production in standard strain (49.19%) as compared to controls (Fig. 6).

### The tested agent’s downregulated the expression of QS-regulatory genes using qRT-PCR

**Fig. 7:**
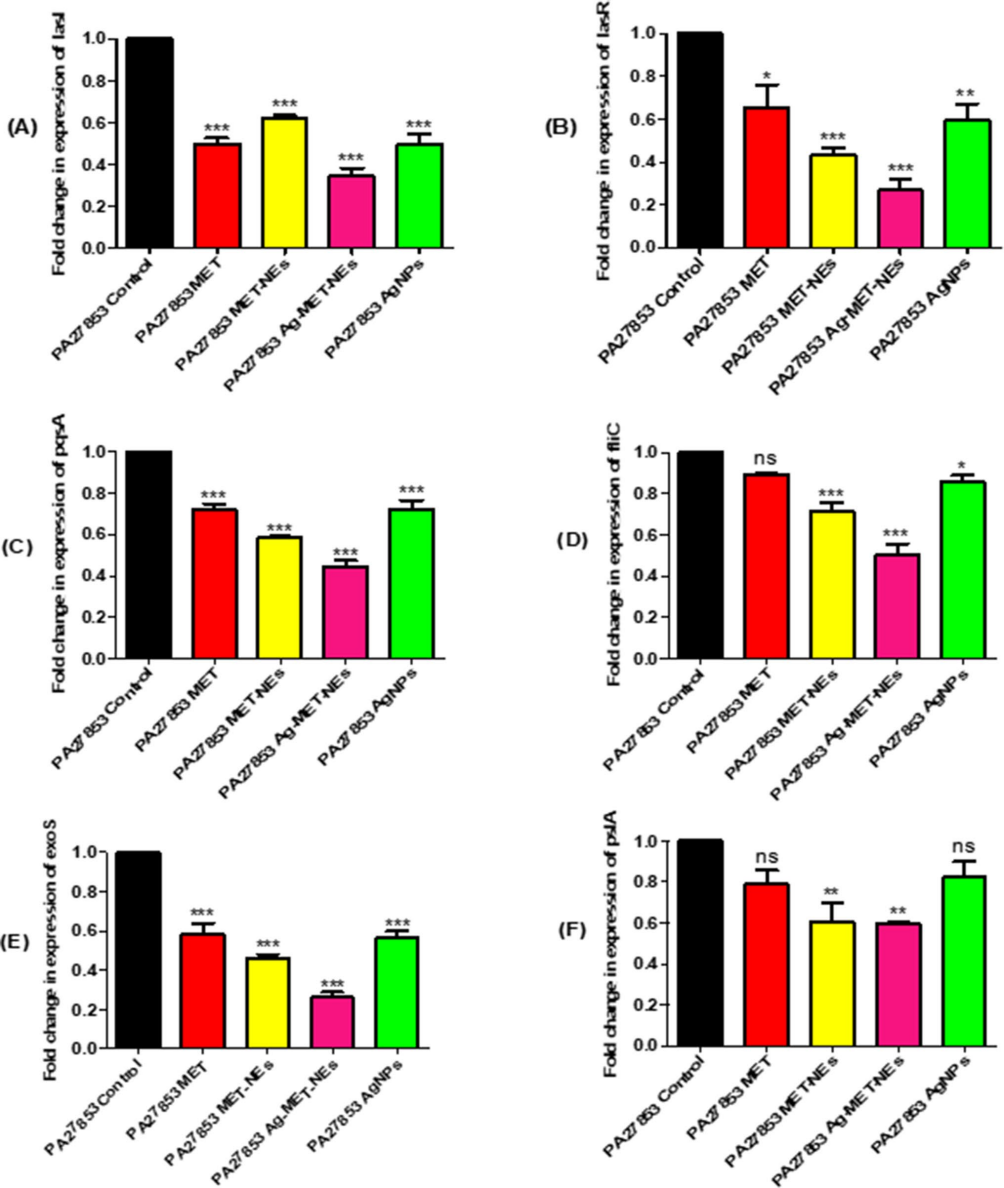
RT-qPCR showed reduced expression of **A)** *lasI*, **B)** *lasR*, **C)** *pqsA*, **D)** *fliC*, **E)** *exoS* and **F)** *pslA* with the tested agents in sub-MICs compared to untreated controls. The data shown are the means ± standard errors of three biological experiments with three technical replicates each. *Significant *P* < 0.05, ns non-significant

The influence of the tested agents on the relative expression of the genes that regulates the virulence factors’ production in the standard strain *P. aeruginosa* ATCC 27853 strain was assessed using qRT-PCR and the results were analyzed via the 2^−ΔΔCt^ method. The expression levels of *lasR*, *lasI*, *pqsA*, *fliC*, *exoS* and *pslA* were significantly decreased after treatment with sub MICs of the tested agents compared to controls (Fig. 7). The expression level of *lasI* gene was reduced significantly; 37.58% with MET-NEs, up to 50% with either MET or AgNPs, while the highest percentage reduction was 65.45% with Ag-MET-NEs. With regards to *lasR* gene expression, the percentage reduction of MET was somehow comparable to that of AgNPs (34.62% and 40.38%), respectively, Ag-MET-NEs exhibited higher reduction (73.08%) than MET-NEs (56.62%). In addition, the expression level of *pqsA* gene was also significantly reduced; MET-NEs and Ag-MET-NEs showed higher reduction (41.56% and 55.41%), respectively than either MET or AgNPs that exhibited lower activities approximatey (27%) each. Moreover, Ag-MET-NEs showed the highest reduction in the expression of *fliC* gene (49.63%), however, MET-NEs (28.89%) and AgNPs (14.07%) had lower reduction. No significant reduction was observed after MET treatment. Furthermore, significant reduction in *exoS* gene expression was observed with MET, AgNPs and MET-NEs (41.67%, 43.75%, and 54.17%, respectively) with the highest reduction found with Ag-MET-NEs (73.84%). Concerning the relative expression of *pslA*, it was significantly diminished with MET-NEs and Ag-MET-NEs nearly (40%) each. However, no significant reduction was found with MET and AgNPs.

### The tested agents decreased the bacterial load in liver and kidney tissues

**Fig. 8:**
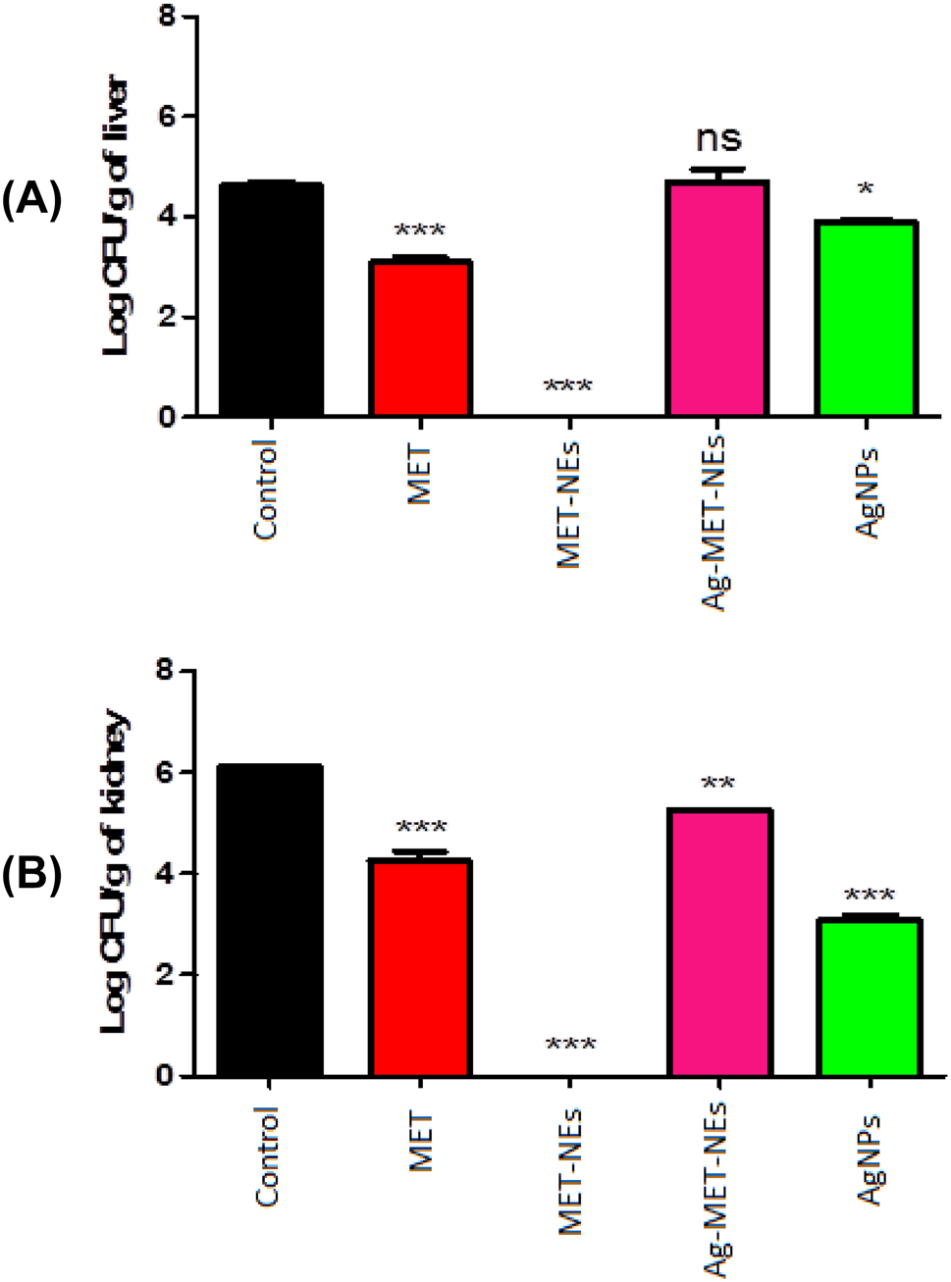
*P*. *aeruginosa* ATCC 27853 CFUs recovered from mice tissues **a** for livers and **b** for kidneys) 24 h post-infection. Bars represent mean log CFUs per gram of organ tissue. The bacterial load was calculated and expressed as means ± standard errors. *Significant *P* < 0.05, ns non-significant

To further study the anti-virulence activities of the tested agents, the bacterial load in livers and kidenys were estimated in the presence and absence of sub MICs of the tested agents using mice as an infection model. The live bacterial counts in the liver and kidney tissues of the mice treated groups were significantly lower than those of the untreated mice group (*P* < 0.05). The results were expressed as log CFU reduction in viable counts per gram of organ tissue. It was found that as compared to MET-NEs that completely eradicated bacteria in liver tissues, the mean log CFU reductions of viable counts decreased from 4.625 in untreated group to 3.090 and 3.895 in MET and AgNPs treated groups, respectively, while no significant reduction of the bacterial burden was observed with Ag-MET-NEs treated group (4.685). Similarly, MET-NEs successfully removed all live bacteria from kidney tissues, surpassing MET, Ag-MET-NEs and AgNPs with mean log CFU reductions from 6.120 in untreated group to 4.255, 5.265 and 3.080, respectively (Fig. 8).

## Discussion

Antibiotic resistance has become a major health problem (Cegelski et al. 2008; Defoirdt 2018). Additionally, there has been a lack of novel antibiotic discoveries in the past decades (Ventola 2015). Antibiotic-resistant pathogens cause serious infections, this is considered as a major reason of morbidity and mortality; therefore new policies are required to tackle this problem such as drug repurosing (Prestinaci et al. 2015; Rangel-Vega et al. 2015; Thangamani et al. 2015).

*Pseudomonas aeruginosa* is a member of *ESKAPE* pathogens that include *Pseudomonas aeruginosa*, *Klebsiella pneumoniae*, *Staphylococcus aureus*, *Enterococcus faecium*, *Enterobacter* spp., and *Acinetobacter baumannii*, that are the leading cause of hospital acquired infections worldwide and possess potential drug resistance mechanisms (Santajit and Indrawattana 2016). Bacteria possess several virulence factors that enable them to infect their hosts and are quorum sensing (QS) regulated (Grandclement et al. 2016; LaSarre and Federle 2013). As a result, QS is considered as an attractive target for anti-virulence treatment.

Nanoparticles are widely used in different applications among which is treatment of bacterial infections and biofilms such as silver nanoparticles (Murphy et al. 2015). Silver nanoparticles (AgNPs) have superior bactericidal activity over Ag^+^ against both Gram negative and Gram positive bacteria (Kora and Arunachalam 2011; Kvítek et al. 2008; Martínez-Castañon et al. 2008). The likelihood of resistance to Ag is assumed to be low. Therefore, silver (Ag)-based compounds have seen a revival (Feng et al. 2000). Moreover, many nanomaterials were found to have anti-virulence activity against *P. aeruginosa* (Pham et al. 2019; Shah et al. 2019; Singh et al. 2015).

In the present study, the quorum sensing and virulence inhibitory activities of the tested agents were investigated against *P. aeruginosa* and using 1/10 MICs to avoid effect on bacterial growth and ensure that inhibitory effect on the tested bacteria is due to anti-virulence activity rather than killing them.

In the current study, the size of Ag-MET-NEs measured in DLS technique is larger than that of AgNPs and MET-NEs. This may be attributed to the hydrophilic properties of Ag-MET-NEs and surrounding molecules. This can be explained in the light of the fact that the particle size is not related only to the metallic NPs’ core, as all adsorbed materials on the surface of NPs such as stabilizers also have an impact on NPs’ particle size. Moreover, the particle size may be affected by the thickness of the electrical bilayer that moves along the nanoparticles (El-Batal et al. 2020).

The best approach of testing the nanoemulsion stability can be accomplished by validation of the particle size in addition to zeta potential along the time. Slight variations in these properties can be detected using an applicable technique, such as DLS.

On testing the Ag-MET-NEs, they were found to be stable. This can be obtained when the values of zeta potential are high (above − 30 mV and less than 30 mV). This ensures that high energy barriers were created to prevent coalescence of the dispersed droplets (Zhao et al. 2010). Our results showed no phase separation or other signs that denote samples′ instability. It is noteworthy that even the particle size changes found in the initially prepared or stored samples were of no significance. Moreover, the stability of the particles of nanoemulsions that are sufficiently small can be correlated with the zeta potential. As previously mentioned, high zeta potential of nanoparticles confers their stability, while low zeta potential denotes that the emulsions will break out and flocculate because of the higher attraction forces than the repulsive ones of the emulsion (Jadhav et al. 2015).

The DLS size ranges of Ag-MET-NEs were higher than measurements by TEM. This can be explained by the fact that DLS measurments are confined or restricted to the NPs′ hydrodynamic diameters. The larger size of NPs measured may be due to the encasement of amphiphilic nanoparticles within water molecules. TEM measures the actual diameters of the nanoparticles (Bendary et al. 2021; El-Batal et al. 2021).

In the current study, it was found that MET-NEs showed similar antibacterial activity to MET against *P. aeruginosa*. However, the combination of MET and AgNPs (Ag-MET-NEs) exhibited high synergistic activity that than that of MET-NEs or AgNPs alone (Table 2). Similar result was observed by Li et al*.* (2020), who compared between the antibacterial activity of biguanide-based polymetformin (PMET) and its nanoform FTP NPs which made from F-127 surfactant, tannic acid and PMET and found that both showed similar activity against the tested bacteria. In addition, Polyhexamethylene biguanide (PHMB), a cationic biocide functionalized silver nanoparticles were tested for their antimicrobial activity against *E. coli*, it was found that PHMB enhanced the antimicrobial properties of AgNPs of about 100 times compared to the previous reports of AgNPs (Ashraf et al. 2012). Another study done by Yi et al*.* reported better bactericidal effect of AgNPs-PHMB as compared to AgNPs and PHMB against *S. aureus* (Yi et al. 2019).

The combined action of the O/W MET-AgNEs, we recommend that, the silver nanoparticles entered into the oily phase and coated by metformin moiety through physical interaction improves release of drug into the target site, and the nano emulsion improved the antibacterial and anti-biofilm activities against different organisms (Prakash et al. 2020) and inactivated the microorganisms more than the standard (Pathania et al. 2018). The nano emulsion systems promote their interaction with the microbial cell membranes by four main routes (Mosallam et al. 2021b); (1) the augmented extent and transport through the outer plasma membrane that increases the interaction with the cytoplasmic membranes; (2) the fusion of the emulsifier droplets with the phospholipid bilayer of the cell membrane that likely promotes the targeted release of the essential oils at the required sites; (3) the sustained release over time of the essential oils from the nano emulsion droplets, driven by essential oils partition between the oil droplets and also the aqueous phase, that prolongs the activity of essential oils; and (4) the electrostatic interaction of charged nano emulsions droplets with charged microbial cell walls that increases the concentration of essential oils at the positioning of action.

*Pseudomonas aeruginosa* has the capability to form biofilm that confers resistance to antibiotics by up to one thousand fold more than planktonic cells which has a major role in bacterial resistance and pathogenesis (Loo et al. 2014; Mah and O’Toole 2001). The incapability of the antimicrobials to penetrate the biofilm matrix (one of the main causes of bacterial resistance) could be overcome via using nanostructures showing anti-biofilm activity (Ansari et al. 2014; Shah et al. 2013). In the present study, it was found that MET-NEs or Ag-MET-NEs demonstrated synergistic activities as they were more effective than either MET or AgNPs alone. Abbas et al*.* (2017) reported higher percentage reduction of PAO1 biofilm by metformin (67.9%). Metformin also enhanced gold nanoparticles′ antibacterial activities and biofilm eradication (Rasko and Sperandio 2010). A study done by Li et al*.* (2020) reported that FTP NPs surpassed PMET with ∼ 100-fold (∼ 2log_10_) greater reduction of MRSA USA300 biofilm bacterial cell counts. In addition, several studies reported antibacterial and anti-biofilm activities of Polyhexamethlene biguanide (PHMB) against a variety of bacterial species (Kamaruzzaman et al. 2017; Lefebvre et al. 2018). Moreover, the studies on another biguanide compound chlorhexidine showed contradictory results. Abdallah and Abakar (2017) reported that chlorhexidine significantly reduced *S. aureus* biofilm depending on the contact time and concentration used, while in another study, chlorhexidine showed no bactericidal effect on *S. aureus* biofilm (Vestby and Nesse 2015). Furthermore, biosynthesized AgNPs reduced the *P. aeruginosa* PAO1 biofilm by < 70% as reported (Hussain et al. 2019; Qais et al. 2020, 2021). On the other hand, Yang and Alvarez (2015) reported that exposure of *P. aeruginosa* PAO1 to non-lethal polyvinylpyrrolidone-coated silver nanoparticles concentration led to increased biofilm formation, enhanced extracellular polymer substances, lipopolysaccharide biosynthesis, and upregulation of antibiotic resistance genes.

The redox active pyocyanin pigment enables *P. aeruginosa* to penetrate host cell membranes and interfere with host cellular functions leading to cellular damage (Hall et al. 2016). In the current study, the lowest inhibition of pyocyanin pigment was found with MET. However, the activity was greatly enhanced using their nanoform. No significant difference between Ag-MET-NEs and AgNPs was observed. This can be attributed to some kind of chemical interaction between MET and AgNPs in the combination (Ag-MET-NEs). Surprisingly, AgNPs increased pyocyanin production in some tested bacteria. Metformin showed higher reduction in pyocyanin pigment in a study reported by Abbas et al*.* (2017). Several studies showed much higher inhibition by biosynthesized AgNPs than the current study such as Qais et al*.* (2020, 2021)However, Ellis et al*.* (2018) reported that *P. aeruginosa* can resist AgNPs by producing phenazine pigments (pyocyanin, pyoverdine, and pyochelin). Pyocyanin can reduce Ag^+^ to Ag^0^, thus it protects the bacteria from the damage caused by silver ions emitted from nanoparticles. Similarly, this result was also in good agreement with Muller and Merrett (2014).

Quorum sensing-controlled swarming motility is essential for *P. aeruginosa* pathogenesis as well as biofilm formation (Pamp and Tolker-Nielsen 2007). In the current study, MET-NEs was the most potent inhibitor of swarming motility as well as biofilm formation, suggesting a role of swarming motility in the biofilm interruption, which is in agreement with Shah et al. (2019). MET-NEs successfully blocked swarming motility more than MET, whereas Ag-MET-NEs and AgNPs similarly impaired bacterial swarming. Hussain et al*.* (2019) reported higher inhibition of *P. aeruginosa* PAO1 swarming motility using biosynthesized silver nanoparticle.

Proteases degrade immunoglobulins and fibrin as well as they disrupt epithelial tight junctions (Kipnis et al. 2006). In the current study, the inhibition of proteolytic activity using MET-NEs surpassed that of MET alone. Similarly, the inhibitory activity of Ag-MET-NEs was more than AgNPs alone. In a study reported by Abbas et al*.* (2017), metformin was capable of reducing the proteolytic activity, which was consistent with the current study. In recent studies done by Qais et al*.* (2020, 2021), biosynthesized AgNPs caused higher percentage reduction of *P. aeruginosa* PAO1 exoprotease activity than the present study.

*Pseudomonas aeruginosa* possesses a unipolar flagellum, which is composed of a polymer of flagellin protein subunits, encoded by the *fliC* gene. It is responsible for mobility and chemotaxis, in addition to helping in the attachment of the bacterium to host cells and non-living surfaces, which augments the ability to colonize and invade during the earlier stages of infections (Haiko and Westerlund-Wikstrom 2013). *P. aeruginosa* also produces exoS, a major cytotoxin implicated in stages of colonization, invasion and dissemination during infection (Bradbury et al. 2010). The three exopolysaccharides namely; Pel, Psl, and alginate have important roles in surface attachment, biofilm formation and stability. The *pslA* gene encodes psl (Billings et al. 2013; Ghafoor et al. 2011).

In the current study, the genes tested are the quorum sensing encoding genes. quorum sensing is the mechanism of cell-to-cell communication. Each cell secretes a molecule of autoinducers, so the concentration of the autoinducers is proportional to the cell density. When, the concentration of autoinducers reaches a certain threshold value, this reflects that the cell density or population reaches the quorum. At this point, the virulence genes are activated. The decrease in the expression of quorum sensing genes means that the tested agents downregulated these genes, and as a result, the production of virulence factors will be reduced. This is a confirmation of the results of the phenotypic investigation.

The expression levels of QS regulating genes *lasR, lasI, pqsA, fliC, exoS* and *pslA* were assessed by qRT-PCR. It was found that the tested inhibitors significantly downregulated the expression levels of such QS regulatory genes **(**Fig. 7**).** Ag-MET-NEs was the most potent inhibitor against QS regulatory genes followed by MET-NEs, and both were more effective than either the bulk MET or AgNPs, which had relatively similar QS inhibitory activity. This confirms that the quorum quenching activity of the nanoform surpassed that of its bulk one and also suggests some kind of synergism between MET and AgNPs in the combination (Ag-MET-NEs). Hegazy et al*.* (2020) reported that the expression levels of *rhlI/R, lasI/R and pqsA/R* was decreased using sub-MIC of metformin. Silver nanoparticles downregulated the expression of *rhlI/R* and *lasI/R* through inhibition of *rhlR* and *lasR* (Singh et al. 2015). Also, Mahnaie and Mahmoudi (2020) discovered that glutathione-stabilized silver nanoparticles exhibited antibiofilm activity in *P. aeruginosa* via lowering the *lasI/R* expression. In addition, Abdelraheem and Mohamed (2021) found that, except for the *toxA* gene, all biofilm and virulence genes of *P. aeruginosa* clinical isolates were significantly downregulated after ZnO NPs treatment. On the other hands, Costabile et al*.* (2015) reported that the QS inhibitory activity of the anthelmintic drug niclosamide (NCL) formulated as nanosuspensions (T80_10 or T80_10 DP) was equivalent to that of the unformulated NCL predissolved in DMSO. However, there are other systems required for *P. aeruginosa* virulence such as two compartment system (Francis et al. 2017).

For the in vivo study, it was found that drugs with anti-virulence activity reduced the colonization rates of invading pathogens as it aided the immune system in eradicating the infection. Regarding the log CFU reduction in viable counts, mice group treated with MET-NEs completely eliminated the living bacteria from livers and kidneys of sacrificed mice, being the most potent among the tested inhibitors; its bulk form (MET), AgNPs and even Ag-MET-NEs combination which may require further research. In addition, the formulation of metformin in nanoform has much lower accumulation than its bulk form or metal nanoparticles, thus reducing cytoxicity that occurs after exposure. Similarly, NCL formulated as nanosuspensions had lower toxicity in a rat lung infection model involving *P. aeruginosa* (Costabile et al. 2015).

In the current study, on the contrary to MET that caused approximately 30% clearance of *P. aeruginosa* ATCC 27853 infection from collected mice tissues, Hegazy et al*.* (2020), reported that metformin failed to protect mice from *P. aeruginosa* PAO1 (ATCC BAA47B1). Escárcega-González et al*.* (2018) reported the capability of AgNPs to reduce CFUs in a murine skin infection model in rats caused by a clinical strain of *P. aeruginosa* as compared to the untreated group.

## Conclusion

In conclusion, targeting bacterial virulence and QS offers an alternative strategy because it curbs the bacterial ability to harm the host rather than affecting their growth, and reduces the emergence of MDR pseudomonal infections. This benefit can be maximized via repurposing of FDA approved medications. Metformin is FDA approved antidiabetic drug with QS inhibitory activity against MDR *P. aeruginosa*. In the present study, it was found that the formulation of metformin in nanoform was promising because it exhibited distinct physical, chemical and bioligical properties as compared to its bulk. In addition, the combination between MET and AgNPs showed synergistic antibacterial effect as well as it greatly inhibited the QS regulatory genes of *P*. *aeruginosa.*

## Data Availability

The authors confirm that the data supporting the findings of this study are available within the article.

## Declarations

### Constant of participate and publish

All authors agree to participate and publish.
